# Household fuel use and adverse pregnancy outcomes in a Ghanaian cohort study

**DOI:** 10.1186/s12978-020-0878-3

**Published:** 2020-02-22

**Authors:** Eartha Weber, Kwame Adu-Bonsaffoh, Roel Vermeulen, Kerstin Klipstein-Grobusch, Diederick E. Grobbee, Joyce L. Browne, George S. Downward

**Affiliations:** 1 Julius Global Health, Julius Center for Health Sciences and Primary Care, University Medical Center Utrecht, Utrecht University, Utrecht, The Netherlands; 2 Institute for Risk Assessment Science (IRAS), Division of Environmental Epidemiology (EEPI), Utrecht University, Utrecht, The Netherlands; 3 Department of Obstetrics and Gynaecology, School of Medicine and Dentistry, University of Ghana, Accra, Ghana; 4 Division of Epidemiology & Biostatistics, School of Public Health, Faculty of Health Sciences, University of the Witwatersrand, Johannesburg, South Africa

**Keywords:** Cooking fuel, Perinatal mortality, Apgar score, Pregnancy outcomes, Perinatal outcomes

## Abstract

**Background:**

Accruing epidemiological evidence suggests that prenatal exposure to emissions from cooking fuel is associated with increased risks of adverse maternal and perinatal outcomes including hypertensive disorders of pregnancy, low birth weight, stillbirth and infant mortality. We aimed to investigate the relationship between cooking fuel use and various pregnancy related outcomes in a cohort of urban women from the Accra region of Ghana.

**Methods:**

Self-reported cooking fuel use was divided into “polluting” (wood, charcoal, crop residue and kerosene) and “clean” fuels (liquid petroleum gas and electricity) to examine 12 obstetric outcomes in a prospective cohort of pregnant women (*N* = 1010) recruited at < 17 weeks of gestation from Accra, Ghana. Logistic and multivariate linear regression analyses adjusted for BMI, maternal age, maternal education and socio-economic status asset index was conducted.

**Results:**

34% (*n* = 279) of 819 women with outcome data available for analysis used polluting fuel as their main cooking fuel. Using polluting cooking fuels was associated with perinatal mortality (aOR: 7.6, 95%CI: 1.67–36.0) and an adverse Apgar score (< 7) at 5 min (aOR:3.83, 95%CI: (1.44–10.11). The other outcomes (miscarriage, post-partum hemorrhage, pre-term birth, low birthweight, caesarian section, hypertensive disorders of pregnancy, small for gestational age, and Apgar score at 1 min) had non-statistically significant findings.

**Conclusions:**

We report an increased likelihood of perinatal mortality, and adverse 5-min Apgar scores in association with polluting fuel use. Further research including details on extent of household fuel use exposure is recommended to better quantify the consequences of household fuel use.

**Study registration:**

Ghana Service Ethical Review Committee (GHS-ERC #: 07–9-11).

## Plain English summary

Three quarters of Ghana’s households use polluting fuels such as wood, kerosene and charcoal for cooking or heating. The household air pollution (HAP) generated from this fuel use can lead to diseases in the lungs, brain and heart. As generally women are responsible for cooking and childcare, they are disproportionately exposed to pollution from cooking fuels. Past research has shown that being exposed to HAP while pregnant can have negative effects on both mother and child. However, very few studies have been published that investigate household fuel use and birth outcomes in sub-Saharan African countries like Ghana (the bulk of studies thus far conducted have been instead based in Asia). It is valuable to understand health risks within local contexts because local evidence may be more persuasive when policy making. We examined the effect of household fuel use on pregnancy outcomes in a cohort of pregnant Ghanaian women. In our cohort we found that women who used polluting fuels had on average lower Apgar scores, a measure to summarize newborn health, after 5 min for babies born as well as higher rates of perinatal mortality (infant death during delivery or within a few weeks after birth).

## Introduction

Globally, an estimated 7 million people die from exposure to indoor and outdoor air pollution [1]. A major source of both indoor (i.e. household) and outdoor air pollution is the domestic combustion of solid fuels (wood, coal, etc.) and kerosene (collectively referred to here as “polluting” fuels) for daily heating and cooking. Over 3 billion people world-wide use polluting fuels in their homes, primarily from low and middle income countries [2, 3].The household air pollution (HAP) generated by cooking fuel use has been estimated to cause 3.8 million premature deaths in 2016, which outnumbers deaths attributed to human immunodeficiency virus (HIV), tuberculosis (TB), and malaria combined [1, 3, 4]. Additionally, since women frequently carry out the duties of gathering fuel and performing cooking [5], the health effects of HAP disproportionately affect women and their accompanying children [3, 5]

Accruing epidemiological evidence suggests that (in-utero) exposure to both indoor and outdoor air pollution during pregnancy is associated with increased risks of pregnancy induced hypertensive disorders [6], low birth weight [7], stillbirth [8, 9] and neonatal death [10]. Inhaled particulate matter can diffuse into the maternal bloodstream causing inflammation, oxidative stress and cell damage [2]. Several studies have reported placental dysfunction resulting from the binding of polycyclic aromatic hydrocarbons (PAH) to DNA [11–15]. These factors may also impair the transport of nutrients to the fetus across the placenta, which can result in low birthweight [4]. Previous literature suggests that polluting cooking fuel use is associated with (pre-)eclampsia [16], low birthweight [17] and stillbirth [8].

There is variability in terms of particle composition, personal cooking behaviours within countries (including within regions of Accra, Ghana) and in between countries [18, 19]. The variability highlights the need to analyze data at regional levels in order to more appropriately address the needs of the target populations for future interventions [19, 20]. Particles emitted vary based on fuel type, stove type, and geography. Geographically, rural and urban dwellers tend to exhibit different behaviours in fuel types used. Generally, charcoal is used more in urban areas and firewood in rural areas [21]. A previous study, investigating fuel usage in Peru, Nepal and Kenya, reported different cooking preferences based on tastes or cultural practices - in Kenya a stove that allowed for personal movement while firmly grounded was preferred to make the staple food ugali, while in Nepal even distribution of heat was important for cooking rice [19].

As of 2016, approximately three quarters (78%) of Ghanaian households use polluting fuels as their main cooking fuel [22]. Further, 14,000 deaths in Ghana are attributed to household air pollution each year [22]. However, there is little research evaluating how this practice impacts Ghanaian women, and much less so on pregnant women. A previous cross-sectional study in Accra, Ghana among women using charcoal or gas stoves reported that charcoal users had lower absolute birthweights (average reduction: 243 g, 95% CI: 496,11) and an elevated risk of low birthweight (Adjusted Risk Ratio (ARR) = 1.41; 95% CI: 0.62,3.23) [23]. The aim of the current study is to examine the relationship between polluting fuel use on multiple pregnancy outcomes in comparison to clean fuel use in a cohort of urban Ghanaian women.

## Methods

### Study design, setting and participants

The current study was a secondary analysis of a prospective cohort study of 1010 pregnant Ghanaian women from within the Accra metropolis. The original purpose of this cohort was to assess the incidence and risk factors of hypertensive disorders of pregnancy. However, we re-used the data to crudely assess household air pollution exposure on various additional pregnancy outcomes in an exploratory manner. Details regarding follow-up and data collection periods are published elsewhere [24, 25]. Briefly, participants were recruited from the outpatient clinics of Maamobi General Hospital and Ridge Regional Hospital in the Greater Accra Region of Ghana between July 2012 to March 2014. Women were eligible for inclusion if they were less than 17 weeks pregnant, over 18 years of age, and had no known history of chronic hypertension. All participants analyzed in this study provided written informed consent, and the study was approved by the Ghana Service Ethical Review Committee (GHS-ERC #: 07–9-11).

### Data sources

Once enrolled, demographic information from the women was collected using a structured questionnaire administered by trained interviewers. Gestational age was obtained from ultrasound-based estimations at antenatal booking (i.e. < 17 weeks gestation) [25]. Outcome data was collected from hospital records and interviews following the end of the pregnancy. Only singleton births were retained for final analysis (see Additional file 1: Fig. S1).

### Cooking fuel use

Cooking fuel use status was determined via the intake questionnaire which asked, “What is the main fuel used for cooking?”. The responses were: firewood/charcoal, kerosene, gas, electricity, crop residue/sawdust and other/specify. Polluting fuel users were defined as those who used firewood, charcoal, kerosene or crop residue/sawdust. Women who used the other listed fuels (gas, electricity) were classified as “clean” fuel users.

### Pregnancy outcomes

The outcomes examined were: mode of delivery, postpartum hemorrhage (PPH), hypertensive disorders of pregnancy (pre-eclampsia, HELLP syndrome or chronic hypertension, with chronic hypertension retrospectively classified if the first elevated blood pressure occurred < 20 weeks gestation), miscarriage, perinatal mortality, pre-term birth, small for gestational age (SGA), low birthweight (< 2500 g), birthweight in grams (continuous), and adverse Apgar score (< 7) after 1 and 5 min. For definitions and diagnostic criteria of the adverse pregnancy outcomes see Additional file 1: Table S1.

### Other variables

Covariates examined to describe the study population were: gestational age at delivery, age (in years) at first antenatal clinic visit, BMI (in kg/m^− 2^), maternal education (no education, primary school, lower secondary/vocational, and upper secondary/professional/tertiary), ethnicity (Akan, Hausa, Ewe and Ga, Ga-Dangme, Mole Dagbon or Gonja, other), parity (1, 2–3, > = 4), socioeconomic status (SES) by asset index quintiles separated into the lowest 40%, middle 40% and highest 20%, and formal employment status. Socioeconomic status by asset was obtained through a previously described principle component analysis based on asset ownership and various household characteristics [24–26].

### Statistical analyses

Bivariate analyses of outcomes by cooking fuel use were calculated for categorical variables using Pearson chi-squared test or Fischer’s exact test when there were fewer than 5 women within a category, and Students T-test for continuous variables. To further analyze the association between cooking fuel use and adverse pregnancy outcomes, multivariate logistic or linear regression models were created. Potential confounders were selected a priori using previous literature on this cohort and then bivariate analysis, resulting in models being adjusted for: socio-economic status, maternal age, maternal BMI, and parity. Complete case analysis was performed, as there was little missing data and data that was missing was considered missing completely at random. When the linearity of the logit assumption was violated (BMI and maternal age) a sensitivity, analysis was conducted by transforming these continuous variables into categorical variables (BMI: < 18, 18 to 24.9, 25–29, and > 30, Maternal age: 18–24, 25-28, 29–31, > 32). All analysis was performed using R version 3.5.2, with the base R package (version 3.6.0), lmtest, and mlogit packages [27–29].

## Results

### Participants

Information on main cooking fuel used was available for all 1010 pregnant Ghanaian women enrolled. Twin pregnancies (*n* = 6), and those with missing outcome data were excluded (*n* = 185), resulting in 819 (82%) women included in the final analysis (Additional file 1: Fig. S1). Overall, the population with missing outcome data did not differ from the study population included in analysis (Additional file 1: Table S3).

### Descriptive data

An overview of the fuel used for cooking is provided in Table 1. The majority of the study population used liquid petroleum gas (LPG) as their main source of cooking fuel (*n* = 270, 65%). The second most utilized fuel was the polluting firewood/charcoal (*n* = 269, 33%). The remaining categories constituted 1% or less of the sample analyzed.

**Table 1:** Main Fuel Used for Cooking ^a^

Fuel Type	N (%)
Firewood/charcoal	269 (33)
Liquid Petroleum Gas	533 (65)
Crop residue/sawdust	1 (< 1)
Kerosene	9 (1)
Electricity	7 (< 1)

^a^ Based-on Miscarriage Outcome where total *n* = 819

Table 2 outlines the socio-demographic characteristics of the study population. The overall mean age at delivery was 28.3 (Standard Deviation (SD) 5.0). Women who used polluting fuels had slightly lower mean BMI [25.0 (SD = 4.6) vs 25.8 (SD = 4.8)]. Nearly all women (79%) had at least lower secondary education. Users of polluting fuels were more likely to have a lower education (19% of polluting fuel users had no education vs. 6% of clean fuel users), and lower SES index (77% in lowest 40% vs. 16.5%). Those who used polluting fuels as their main cooking fuel were less likely to be formally employed (96% vs. 83%).

**Table 2:** Socio-Demographic characteristics of the study population(*N* = 819)

Characteristic		Cooking Fuel Use		
		Polluting (n = 279)	Clean (*n* = 540)	
	Total Mean (SD)	Mean (SD)	Mean (SD)	*P*-value
Gestational Age at Delivery (wks.) (*n* = 732)	39.0 (1.9)	39.1 (1.9)	39.0 (1.9)	0.68
Age at first ANC visit (yr.) (n = 819)	28.3 (5.0)	27.8 (5.6)	28.5 (4.8)	0.06
BMI (kg m^−2^) (*n* = 813)	25.5 (4.8)	25.0 (4.6)	25.8 (4.8)	0.01
	Total group N (%)	N (%)	N (%)	P-value
Maternal Education (n = 819)				< 0.001
No education	83 (10)	52 (19))	31 (6)	
Primary school	96 (12)	40 (14)	56 (10)	
Lower Secondary or Vocational	390 (48)	143 (51)	247 (46)	
Upper Secondary & Tertiary	250 (31)	44 (16)	206 (38)	
SES by asset index (n = 819)				< 0.001
Lowest 40%	303 (37)	217 (77)	86 (16.5)	
Middle 40%	339 (41)	50 (18)	289 (54)	
Highest 20%	177 (22)	12 (4)	165 (30.5)	
Formal Employment (n = 819)				< 0.001
Yes	103 (13)	11 (4)	92 (17)	
No	716 (87)	268 (96)	448 (83)	
Ethnicity (n = 819)				0.03
Akan	293 (36)	97 (35)	196 (36)	
Hausa	158 (19)	57 (20)	101 (19)	
Ewe	171 (21)	48 (17)	123 (23)	
Ga,Ga-Dangme	83 (10)	25 (9)	58 (11)	
Mole, Dagbon, Gonia, Other	114 (14)	52 (19)	62 (11)	
Parity (n = 819)				0.89
0–1	573 (70)	193 (69)	380 (70)	
2—3	230 (28)	81 (29)	149 (28)	
> =4	16 (2)	5 (2)	11 (2)	
Vitamin Use During Pregnancy	411 (50)	174 (47)	326 (50)	0.37

Significant at *P* value < 0.05 for Chi-square test or T-test

### Descriptive characteristics of the pregnancy related outcomes

Table 3 describes pregnancy outcomes by fuel use, where significant differences in miscarriage, perinatal mortality, preterm birth, and low Apgar score 5 min after birth were observed. Of the 39 cases of miscarriage, 19 occurred in those who reported using polluting fuels despite it being the smaller group. For the 11 occurrences of perinatal mortality, 7 occurred among polluting fuel users (a 3% mortality rate), versus 4 among the clean fuel users (1% mortality rate). Of the 29 cases of low Apgar score after 5 min, 16 occurred within the polluting fuel group (6% rate) compared to 13 in the clean fuel group (3%). Other outcomes did not show a significant difference between the two groups. For example, of the 54 preterm births, 20 occurred in the polluting group (rate of 8%) versus 34 in the clean fuel group (rate of 7%).

**Table 3:** Incidence of adverse birth outcomes by household main fuel source

		Cooking Fuel Use		
Outcome	Outcome	Polluting	Clean	
	Total	N (%)	N (%)	P-value
Caesarean Section (*n* = 784)	86	30 (12)	56 (11)	0.72
Post-partum Hemorrhage (*n* = 700)	21	9 (4)	12 (3)	0.35
Hypertensive disorders of pregnancy (*n* = 767)	71	19 (7)	52 (10)	0.21
Chronic Hypertension(*n* = 712)	16	4 (2)	12 (3)	0.59
Miscarriage (n = 819)	39	19 (7)	20 (4)	0.04
Perinatal Mortality (n = 784)	11	7 (3)	4 (1)	0.04^a^
Preterm Birth (*n* = 734)	54	20 (8)	34 (7)	0.56
SGA (*n* = 723)	18	7 (3)	11 (2)	0.63
Birthweight < 2500 (*n* = 772)	86	30 (12)	56 (11)	0.73
Apgar Score < 7 at 1 min (*n* = 750)	115	41 (16)	74 (15)	0.63
Apgar Score < 7 at 5 min (*n* = 748)	29	16 (6)	13 (3)	0.01
	Mean (SD)	Polluting Mean (SD)	Clean Mean (SD)	P-value
Birthweight (n = 772)	3127 (492)	3101 (489)	3140 (494)	0.29

### Findings of regression analysis

Table 4  shows the findings of logistic and linear regression for all outcomes adjusted for BMI, age, SES, and maternal education level. Women who used polluting fuels were at significantly higher odds of experiencing perinatal mortality (OR: 7.6, 95%CI 1.67, 36) than those who did not. The use of polluting fuels was also associated with an Apgar score below 7 that persisted after 5 min (OR: 3.83 95%CI 1.44, 10.11). Directionally positive but non-significant ORs were observed for post-partum hemorrhage (OR: 1.83 95%CI 0.56, 5.95), miscarriage (OR:2.10 95%CI 0.91,4.81), preterm birth (OR:1.01 95%CI 0.48,2.10), and birthweight < 2500 g (OR: 1.05 (0.57,1.93). Sensitivity analysis (Additional file 1: Table S5) found no difference categorizing age and BMI, except for perinatal death which had an attenuated (but still significant) OR compared to that in the main analysis (OR:5.38 95%CI 1.15, 26.15).

**Table 4:** Association between fuel use and birth outcomes

Outcome	Household Fuel Use	
	Polluting^ab^	Polluting^ab^
	Crude OR(95%CI)	Adjusted OR(95%CI)^b^
Caesarean Section	1.09 (0.67–1.73)	1.13 (0.60–2.12)
Post-partum Hemorrhage	1.51 (0.60–3.62)	1.83 (0.56–5.95)
Hypertensive disorders of pregnancy	0.70 (0.39–1.20)	0.79 (0.38–1.62)
Chronic Hypertension	0.64 (0.17–1.87)	0.41 (0.08–1.75)
Miscarriage	1.90 (0.99–3.63)	2.10 (0.91–4.81)
Perinatal Mortality	3.59 (1.07–13.83)	7.6 (1.67–36.0)
Preterm Birth	1.18 (0.65–2.08)	1.01 (0.48–2.10)
SGA	1.26 (0.46–3.25)	1.43 (0.40–4.89)
Birthweight < 2500	1.08 (0.66–1.72)	1.05 (0.57–1.93)
Apgar Score < 7 at 1 min	1.10 (0.72–1.66)	1.12 (0.65–1.92)
Apgar Score < 7 at 5 min	2.51 (1.19–5.41)	3.83 (1.44–10.11)
	Crude β (CI 95%)	Adjusted β (CI 95%)
Birthweight (g)	−39.22(−113.09–34.65)	−4.422(−98.08–89.23)

^a^Vs. Clean (reference) ^b^Adjusted for BMI, maternal age, maternal education and SES

## Discussion

In this study of pregnancy outcomes and household fuel use in Ghanaian women, we observed significantly higher risks of perinatal death and low Apgar score after 5 min among polluting fuel users.

The finding that polluting household fuel use is associated with perinatal mortality is largely consistent with the existing literature. For example, a meta-analysis conducted by Pope et al. who examined four studies from India and Pakistan (representing a total of 33,935 singleton births), reported a pooled OR of 1.51 (95% CI: 1.23,1.85) in association with polluting fuel use [7, 17, 30–32]. A multi-center study conducted by Patel et al. further corroborated our finding reporting an aRR of 1,44 (95%CI: 1.30,1.61). Additionally, a cross-sectional study by Epstein et al. utilizing pooled Ghanaian Demographic and Health survey data from 2003 and 2008 found that the use of polluting household fuels increased the risk of infant mortality (OR = 1.77,95%CI 0.95–3.32) [33]. The elevated likelihood of perinatal mortality may also be reflected in the observed findings for miscarriage where, in the bivariate analysis, a significant relationship between miscarriage and polluting fuel use was observed. However, in the fully adjusted regression model, the relationship between fuel use and miscarriage, while positive, was non-significant (OR: 2.10, 95% CI: 0.91, 4.81). One consideration however is that the rate of miscarriages may be under-estimated as women with miscarriages very early on in their pregnancy would have been unlikely to join the cohort. While we cannot discount that this may be a chance finding, the positive directionality with miscarriage that coincides with the significant and positive perinatal mortality finding suggests that there may be similar underlying mechanisms which deserve further study.

The Apgar score provides an important overview of newborn health, with the score after 5 min tending to be a better predictor of neonatal death than 1 min [34], which may relate to our finding of increased perinatal mortality. A study previously conducted in Nigeria examining factors related to low Apgar score observed that kerosene use was associated with low Apgar score after 1 min. However, to the best of our knowledge, the current paper is otherwise the first study to investigate the independent association between multiple cooking fuels and adverse Apgar scores.

Directionally positive but non-significant OR’s were observed for several other pregnancy outcomes, indicating potential avenues for further exploration. For example, the positive direction of the estimate for low birthweight (OR: 1.08, 95% CI: 0.66, 1.72) is consistent with that reported in the larger multi-center study by Pope et.al. (1.38, 95% CI: 1.25,1.52) [7, 35]. In another study conducted in India, kerosene biomass, and coal fuel use were independently associated with low birthweight (OR for kerosene: 1.51 [1.08,2.12]; OR for biomass 1.24[1.04,1.48]) as was coal use 1.57[1.03,2.41] [10].

Past studies examining hypertensive disorders of pregnancy in relation to wood as cooking fuel are inconsistent, one study showed reduced mean arterial blood pressure with wood use (− 2.0 mmHg; 95% CI: − 3.77, − 0.31), whilst another showed increased risk of self reported symptoms of pre-eclampsia/eclampsia and polluting fuel use (OR = 2.21; 95%: 1.26–3.87; *P* = 0.006) [16]. The latter study may also include those who already had hypertensive disorders before the onset of their pregnancy.

There are several avenues through which the products of polluting fuel combustion (e.g. carbon monoxide [CO], particulate matter [PM_2.5_]) may impact fetal health. Particularly small particles, such as CO may cross the placental barrier where they subsequently act directly upon fetal health and development. For example, CO has a strong affinity for fetal hemoglobin, compromising fetal oxygenation. Additionally, PM_2.5_ either acting systemically or via placental deposition, may cause oxidative stress and an inflammatory response, further impacting fetal development. Animal studies have for example, identified that PM_2.5_ exposure impacts placental morphology and impairs maternal-fetal interaction. It has also been speculated that overly physically demanding work (e.g. collecting firewood) can lead to poor pregnancy outcomes, however the clinical evidence is inconsistent [36–39]. Information on physical exertion within the current study was unfortunately unavailable, however there is an ongoing interventional study investigating the impact of physical exertion on birth outcomes occurring in Ghana which may provide further insights [40].

The strengths of the current study include its prospective design with high retention rates and complete case information. The exploratory nature of this study allowed for a range of outcomes to be analyzed and provides a framework for a more detailed investigation in the future for relevant targeted outcomes. A major limitation of the current study was that exposure information was limited to only questionnaire information about fuel. This means that quantified exposure information and an exposure response relationship is unavailable [40]. Instead, we used the classifications of “polluting” and “clean” fuel to approximate “high” and “low” exposures to indoor air pollution. However, without direct measurements, these classifications are imprecise. Further, it is a common practice for women in Ghana to cook outdoors, meaning that not only their own cooking practices, but those around them are likely to be important for personal exposures. Additionally, information differentiating individual fuel categories (e.g. charcoal/wood) was not available, nor was information on time spent cooking, fuel stacking, fuel quality, ventilation, and stove condition. Methods to combat this in future research would be to collect exposure metrics such as personal exposure monitors, room monitors, or biomarkers and to gain more detailed knowledge of the type of stove, ventilation, hours spent cooking, and fuel moisture [41].

A further limitation is that the small sample size of the current study limits its statistical power, resulting in wide confidence intervals and many suggestive but non-significant findings which, while suggestive, need to be interpreted with caution.

Some of the findings of the current study may be broadly generalizable to urban non-smoking, pregnant women in other urban areas in sub-Sahara Africa, but less so to rural areas outside of the city as differences in behaviours between rural and urban (Ghanaian) populations has previously been reported [21]. Women in Ghana typically do not smoke (smoking rate: 0.4%) and most have access to publicly provided prenatal care visits and vitamin supplementation provided by the National Health Insurance Scheme (NHIS) [42]. The percentage of homes using polluting fuels for cooking (34%) in the current study is slightly lower than the Accra average of 40% [43], which may represent differing health seeking behaviours between the two groups (e.g. users of polluting fuel types may be less likely to attend hospital outpatient clinics – which is where participant recruitment took place). The broader public health implications of cleaner fuel use extend beyond pregnancy and pregnancy outcomes as smoke exposure is linked to a multitude of medical conditions such as respiratory infections, lung cancer, chronic obstructive pulmonary disease, heart disease, and blindness [44]. Furthermore, improvements in air quality have been established to prolong overall life expectancy in the general population [45].

## Conclusions and Recomendations

Polluting cooking fuels are commonly used in urban Ghanaian households leading to avoidable pollutant exposure. In this exploratory study, we observed an association between polluting fuel use and perinatal mortality and an Apgar score below 7 after 5 min. Limited sample size and lack of quantified exposure metrics limit these findings and thus we recommend larger studies incorporating comprehensive assessments of fuel use and environmental exposures to better understand the role of cooking fuels in Ghanaian maternal health. As cooking fuel use is a modifiable risk factor, strategies to improve fuel use and aid in the transitioning of Ghanaians towards cleaner fuel use (e.g. coordinated efforts to ensure reliable LPG access in Ghana) are likely to result in tangible population health gains.

## Supplementary information

**Additional file 1: Table S1.** Definitions of Adverse Pregnancy Outcomes. **Figure S1.** Flowchart Of Those Included From Original Cohort. **Table S3.** original cohort descriptive data of including those lost to follow up (*N* = 185). **Table S5.** Sensitivity Analysis For Selected Outcomes With Linearity Of Logit Assumption Violated.

## Abbreviations

BMI: Body mass index
CH: Chronic Hypertension
CI: Confidence interval
CO: Carbon monoxide
CS: Cesarean section
HAP: Household air pollution
HIV: Human Immunodeficiency Virus
LMICs: Low- and Middle-Income Countries
LPG: Liquid Petroleum Gas
NHIS: National Health Insurance Scheme
OR: Odds ratios
PAH: Polyaromatic Hydrocarbons
PCA: Principle component analysis
PE: Pre-eclampsia
PM: Particulate Matter
PPH: Postpartum hemorrhage
SD: Standard deviation
SES: Socio-economic status
TB: Tuberculosis
WHO: World Health Organization
