# Supplementary material for: Household fuel use and adverse pregnancy outcomes in a Ghanaian cohort study
Source: Reprod Health. 2020 Feb 22;17:29. doi: 10.1186/s12978-020-0878-3 (PMC7036189; doi:10.1186/s12978-020-0878-3)
Supplement: Supplementary file 1 — Additional file 1: Table S1. Definitions of Adverse Pregnancy Outcomes. Figure S1. Flowchart Of Those Included From Original Cohort. Table S3. original cohort descriptive data of including those lost to follow up (N = 185). Table S5. Sensitivity Analysis For Selected Outcomes With Linearity Of Logit Assumption Violated. [file 12978_2020_878_MOESM1_ESM.docx]

**Supplementary Material**

**Cooking Fuel Use and Adverse Pregnancy Outcomes in A Ghanaian Cohort**

Eartha Weber, Kwame Adu-Bonsaffoh, Roel Vermeulen, Kerstin Klipstein-Grobusch, Diederick E. Grobbee, Joyce L. Browne, George S. Downward,

**Table S.1 Definitions of Adverse Pregnancy Outcomes**

| **Outcome** | **Definition** |
| --- | --- |
| Mode of delivery | The mode of delivery was categorized into those who gave birth vaginally or with a caesarean section. |
| Postpartum hemorrhage (PPH) | Primary PPH: more than 500ml of blood loss within 24 hours of delivery for vaginal birth or 1000ml or more for cesarean section. The blood loss was assessed visually by the midwife or doctor who conducted the delivery. Postpartum hemorrhage was considered secondary if it occurred after 24 hours following delivery. |
| Gestational Induced hypertensive disorders (gestational hypertension, pre-eclampsia/eclampsia, HELLP syndrome or chronic hypertension.) | Gestational hypertension was assigned any time after 20 weeks of gestation with either a systolic blood pressure of ≥140 mmHg or diastolic blood pressure of ≥90 mmHg^4^ or both confirmed with two measurements without significant proteinuria^3,4^. Pre-eclampsia was defined as new onset of hypertension and proteinuria of 2+ measured with a urine dipstick occurring after 20 weeks of gestation. Eclampsia was classified as those with pre-eclampsia symptoms with the addition of having tonic-clonic seizures^3,4^. |
| Chronic hypertension | Chronic hypertension was defined as having hypertension that was discovered prior to conception or before 20 weeks of gestation^3,4^. |
| Perinatal mortality | Still birth was defined as fetal death after 24 weeks in utero^5^. Neonatal death was defined as a death which occurred before the first 28 days of life^6,7^. |
| Miscarriage | Miscarriage was defined as loss of the fetus, before its capability for independent survival^6^. |
| Pre-term birth | Preterm birth was defined as children born less than 37 weeks in gestation^6^. |
| Small for gestational age | Small for Gestational Age was defined as birthweight being two standard deviations below the mean for how long the fetus was in the womb^6^. |
| Birthweight | Birthweight was analyzed as both a categorical and continuous outcome. Birthweight was either measured as <2500 grams as low birthweight/normal birthweight, or continuously as its number in grams^6^. |
| Apgar Score | An Apgar score greater than 7 was considered a normal score, while less than 7 was considered an adverse score^6^. |

**Figure S.1 Flowchart of those included from original cohort**


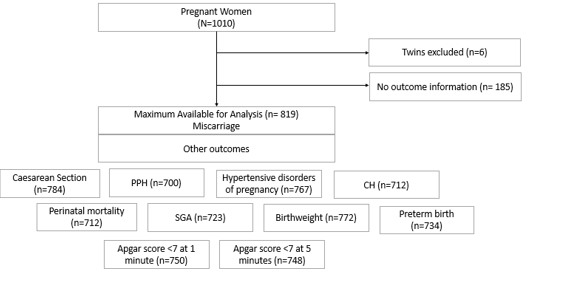


**Table S.3 Original Cohort Descriptive Data of including those lost to follow up (N=185)**

|  |  |  | **Cooking Fuel Use Status** | |
| --- | --- | --- | --- | --- |
| **Characteristic** |  | **Polluting (n=84)** | **Clean**  **(n=101)** |  |
|  | **Total Mean (SD)** | **Mean (SD)** | **Mean**  **(SD)** | **P-value** |
| Age at first ANC visit (yr.) | 26.7 (5.2) | 25.87(5.21) | 27.43(5.03) | 0.04^a^ |
| BMI (kg m^-2^) (n=994) | 24.9 (4.3) | 24.90 (4.57) | 24.86(4.16) | 0.95 |
|  | **Total group N(%)** | **N** | **N** | **P-value** |
| **Maternal Education** |  |  |  |  |
| No education | 27 (15) | 18 (21) | 9 (9) | P<0.001^a^ |
| Primary school | 36 (19) | 22 (26) | 14 (14) |  |
| Lower Secondary or Vocational | 81 (44) | 33 (39) | 48 (48) |  |
| Upper Secondary & Tertiary | 41(22) | 11 (13) | 30 (30) |  |
| **SES by asset index** |  |  |  |  |
| Lowest 40% | 100 (54) | 76 (90) | 24 (24) | P<0.001^a^ |
| Middle 40% | 61 (33) | 7(8) | 54(53) |  |
| Highest 20% | 24 (13) | 1(1) | 23(23) |  |
| **Formal Employment** |  |  |  |  |
| Yes | 19 (10) | 3(4) | 16(16) | 0.006^a^ |
| No | 166 (90) | 81(96) | 85(84) |  |
| **Ethnicity** |  |  |  |  |
| Akan | 64 (35) | 24(29) | 40(40) |  |
| Hausa | 36 (19) | 18(21) | 18(18) |  |
| Ewe | 42 (23) | 18(21) | 24(24) |  |
| Ga,Ga-Dangme | 14 (8) | 7(8) | 7(7) |  |
| Mole,Dagbon,Gonia,Other | 29 (16) | 17(20) | 12(12) |  |
| **Parity** |  |  |  | 0.55^b^ |
| 0-1 | 147 (79) | 66(78) | 81(80) |  |
| 2-3 | 34 (18) | 15(18) | 19(19) |  |
| >=4 | 4 (2) | 3(4) | 1 (1) |  |
| ^a^ Significant at P value <0.05 ^b^ Fischer’s Exact test if group total was < 5 | | | | |

**Table S.5 Sensitivity Analysis for selected outcomes with linearity of logit assumption violated**

|  | **Cooking Fuel Use** |  |
| --- | --- | --- |
| **Outcome** | **Polluting^c^** | **Polluting^c^** |
|  | **OR (95%CI)** | **Adjusted OR(95%CI)** |
| Caesarean Section^a^ | 1.09 (0.67-1.73) | 1.22 (0.65-2.28) |
| Perinatal Mortality | 3.59 (1.07-13.83) | 5.38(1.15-26.15)^a^ |
| Small for Gestational Age^b^ | 1.26 (0.46-3.25) | 0.67(0.18-2.54) |
| ^a^ age was categorized ^b^ age and BMI was categorized ^c^ clean (reference) | | |

Reference

1. Groot, A. D. *et al.* Equity in maternal health outcomes in a middle-income urban setting: a cohort study.

2. Tranquilli, A. L. *et al.* The classification, diagnosis and management of the hypertensive disorders of pregnancy: A revised statement from the ISSHP. *Pregnancy Hypertens. Int. J. Womens Cardiovasc. Health* **4**, 97–104 (2014).

3. Brown, M. A., Lindheimer, M. D., Swiet, M. de, Assche, A. V. & Moutquin, J.-M. The Classification and Diagnosis of the Hypertensive Disorders of Pregnancy: Statement from the International Society for the Study of Hypertension in Pregnancy (ISSHP). *Hypertens. Pregnancy* **20**, ix–xiv (2001).

4. Steegers, E. A. P., von Dadelszen, P., Duvekot, J. J. & Pijnenborg, R. Pre-eclampsia. *Lancet Lond. Engl.* **376**, 631–644 (2010).

5. Nguyen, R. & Wilcox, A. Terms in reproductive and perinatal epidemiology: I. Reproductive terms. *J. Epidemiol. Community Health* **59**, 916–919 (2005).

6. Nguyen, R. & Wilcox, A. Terms in reproductive and perinatal epidemiology: 2. Perinatal terms. *J. Epidemiol. Community Health* **59**, 1019–1021 (2005).

7. Standard Terminology for Fetal, Infant, and Perinatal Deaths | From the American Academy of Pediatrics | Pediatrics. Available at: https://pediatrics-aappublications-org.ezproxy.ub.unimaas.nl/content/137/5/e20160551. (Accessed: 3rd July 2019)
